# Factors Affecting Bilirubin Levels during First 48 Hours of Life in Healthy Infants

**DOI:** 10.1155/2013/316430

**Published:** 2013-06-06

**Authors:** Betul Siyah Bilgin, Ozge Altun Koroglu, Mehmet Yalaz, Semra Karaman, Nilgun Kultursay

**Affiliations:** ^1^Division of Neonatology, Department of Pediatrics, Ege University Faculty of Medicine, Izmir, Turkey; ^2^Department of Anesthesiology and Reanimation, Ege University Faculty of Medicine, Izmir, Turkey

## Abstract

*Objective*. To investigate the relationship of delivery type, maternal anesthesia, feeding modalities, and first feeding and meconium passage times with early bilirubin levels of healthy infants. *Methods*. Cord, 24 hours' and 48 hours' total bilirubin levels were measured in 388 study infants. *Results*. Infants born with cesarean section were fed later and more often had mixed feeding. First meconium passage was delayed with general anesthesia. Cord, 24 and 48 hours' bilirubin levels were not correlated with first feeding time, meconium passage time, mode of delivery, existence and type of anesthesia, and feeding modalities. Being in high intermediate risk zone at 72 hours of Bhutani's nomogram was only related to first feeding time and high cord bilirubin level. Late preterm infants were more frequently born with cesarean section and offered supplementary formula. Therefore, first meconium passage times and bilirubin levels were similar in the late preterm and term infants. *Conclusions*. Type of delivery or anesthesia, late prematurity, feeding modalities, and first meconium passage time were not related to early bilirubin levels in healthy neonates, but delayed first feeding and high cord bilirubin levels were related to be in higher risk zone for later hyperbilirubinemia.

## 1. Introduction

Despite being a temporary condition, neonatal jaundice is still the most common cause of hospitalization in the first week of life. Physiological jaundice is due to the developmental insufficiency of bilirubin uptake, transport, and conjugation in the newborn liver. Decreased gut motility, delayed passage of bilirubin rich meconium, and absence of intestinal bacteria that degrades bilirubin to urobilinogen may all contribute to hyperbilirubinemia by increasing enterohepatic circulation [1, 2]. Delayed initiation of breastfeeding and technical problems in nursing may cause maternal milk insufficiency and increase the risk of hyperbilirubinemia [2].

In recent years, increased cesarean section (c/s) rates and promotion of breastfeeding and earlier hospital discharge caused an increased frequency of neonatal hyperbilirubinemia [3, 4]. Jaundice is more prominent and lasts longer in breastfed infants [1]. Type of the anesthesia used for c/s may also affect the risk for hyperbilirubinemia [5].

Understanding the major contributing factors of early neonatal hyperbilirubinemia may help clinicians to identify the infants at highest risk of hyperbilirubinemia since earlier discharge is a new cost reducing strategy in most clinics. Furthermore, identifying infants with risk of hyperbilirubinemia and observing closely before discharge might reduce both morbidity and readmission rates [3].

Therefore, the relation of gestational age, delivery type, existence and type of maternal anesthesia, feeding modalities, and first meconium passage time with early bilirubin levels of healthy infants was investigated in this study. As a secondary aim we planned to analyze the complex relationship among these parameters.

## 2. Methods

This prospective study was carried out in Ege University Faculty of Medicine, Department of Obstetrics and Gynecology, between November 2011–February 2012 in infants born after 34 weeks of gestational age. The study was approved by our institutional Ethics Committee (12. Nov. 2011 ID: B.30.2.EGE.0.20.05.00/OY/1413/569). Infants with severe problems like congenital anomaly, asphyxia, traumatic birth caused by *vacuum extraction*, respiratory insufficiency, infection, metabolic diseases, and hemolytic diseases (i.e., Rh and ABO incompatibility) not only with the positive Coombs tests but also with other criteria for hemolysis—such as lowering hematocrit levels *reticulocytosis* and peripheral blood smear—were excluded from the study. Gestational ages were determined according to the last menstrual period or with Dubowitz score if last menstrual period was not known [6]. Small for gestational age (SGA), appropriate for gestational age (AGA), and large for gestational age (LGA) were defined according to the Lubchenco percentiles [7].

Our hospital is a baby-friendly hospital with approximately 3000 deliveries per year. Breast feeding is encouraged for all infants born in our hospital. Infants born with vaginal route following an uncomplicated delivery are discharged at 24 hours of life, while infants born with c/s are discharged at 48 hours of life.

Cord blood and 24 hours' total bilirubin levels of all study infants and additionally 48 hours' bilirubin levels in babies who stayed up to 48 hours at the hospital were measured. Total bilirubin levels were measured in capillary blood by bilirubinometer (Bilimeter 3 Pfaff Medical) with spectrophotometric method. Delivery and anesthesia types, Apgar scores, birth weights, birth lengths and head circumferences, gender, first feeding and first meconium passage times, and blood groups of mothers and infants were recorded. According to their feeding modality, infants were classified into three groups as breastfed, formula-fed, and breastfed with formula support (mixed feeding).

Routine anesthesia practices of our clinic are 10–12 mg 0.5% hyperbaric bupivacaine (Marcaine Heavy) (total volume 2 ± 0.3 mL) for spinal anesthesia and 0.5% levobupivacaine (Chirocaine) (total volume 18 ± 8 mL) as 1–1.5 mL of volume per segment for epidural anesthesia. During regional anesthesia, patients receive volume replacement with 15 mL/kg crystalloid solution to prevent maternal hypotension, and if systolic blood pressure is ≤90 mmHg intravenous (i.v.) 5 mg ephedrine is administered until systolic blood pressure rises above 100 mmHg. If maternal heart rate is ≤55 beats/min, 0.5 mg atropine i.v. is administered. For general anesthesia practices, induction is performed by 10 *μ*g/kg atropine, 4-5 mg/kg thiopental sodium, 0.6 mg/kg rocuronium, and maintenance is provided by inhaled 50% O_2_ + room air and 1-2% sevoflurane.

Data were analyzed by using SPSS for Windows 15.0 software package. Results were given as Median/Interquartile Range (IQR), and *P* values lower than 0.05 were accepted as significant. Normal distribution of data was checked through the Shapiro-Wilk test, and since data distribution was found inappropriate for normal distribution, nonparametric tests were chosen. Spearman's correlation analysis was used for correlations. Mann-Whitney test was used for determining the differences between two independent groups. Repeated measurements (cord, 24 and 48 hours' bilirubin levels) were analyzed with variance analysis. Fisher's exact test, *Kruskal-Wallis*, or Chi-square tests were used for categorical data analysis. 

## 3. Results

Demographic data of 388 infants enrolled in the study are summarized in Table 1. Median and (IQR) values for gestational age, birth weight, first meconium passage, and time for first feeding time were found as 38 (2) weeks, 3150 (695) grams, 6 (7) hours, 1 (1) hours, respectively, in study groups. Breastfeeding alone, breastfeeding plus formula supplementation (mixed feeding), and formula feeding were the feeding modalities in 58.5%, 41%, and 0.5% of infants, respectively (Table 1). Without hemolytic disease, the rate of Rh incompatibility was found as 6.44% (*n* : 25), and the rate of ABO blood group incompatibility was found 23.96% (*n* : 93) (Table 1).

The relationship of delivery modes, anesthesia types, first feeding time, meconium passage time, feeding types, and bilirubin levels is shown in Table 2. 

Initiation of first feeding was significantly later in infants born with c/s (1.5 (1) hours) than infants born vaginally (0.5 (0.5) hours) (*P* = 0.00). Vaginally born infants were more frequently breastfed (*P* = 0.00). Meconium passage was significantly later in infants born after general anesthesia (thiopental) compared to spinal anesthesia (hyperbaric bupivacaine) and epidural anesthesia (levobupivacaine) (*P* = 0.00). First meconium passage time was not correlated with first feeding time and feeding types. Bilirubin levels were not correlated with delivery modes, anesthesia types, first meconium passage time, first feeding time, and feeding types (Table 2).

Data obtained from late preterm and term infants in comparison of delivery modes, anesthesia types, feeding modalities, first meconium passage time, cord, 24th hour and 48th hour bilirubin levels and the frequency of 24th hour bilirubin level higher than 6 mg/dL are shown in (Table 3). This high incidence of c/s is due to the increased c/s rates according to mother's will in our country similar to the trends in the world [8]. Another reason is that our hospital is a regional reference center for the high risk deliveries often requiring c/s. For this reason, the c/s rate was significantly higher in late preterm infants (81.7%) than in term infants (69.4%) (*P* = 0.01). Late preterm infants were less frequently exclusively breastfed (39.2%) than term infants (67.2%) (*P* = 0.00). However, there were no significant differences between the groups regarding first meconium passage time, cord blood, 24 and 48 hours' bilirubin levels and being in the high intermediate risk zone (serum bilirubin level higher than 6 mg/dL) on Bhutani's nomogram [9] at 24 hours' (Table 3). 

Evaluation of hyperbilirubinemia risk with the predictive value of 24 hours' bilirubin level higher than 6 mg/dL on Bhutani's nomogram showed that this risk increase was only related to the first feeding time and increased cord blood bilirubin levels (*P* = 0.03, *P* = 0.00, resp.). (Not shown in tables.)

## 4. Discussion

Hyperbilirubinemia and related complications can be prevented by early recognition of risk factors and close follow-up of high risk infants [3]. Therefore, this study aimed to find out these risk factors in healthy infants. It may consider that cord clamping time may increase rates of hyperbilirubinemia together with increased hematocrit levels. However, delayed cord clamping—for more than a minute—has never been proven to increase the rate of neonatal symptomatic disease. In a systematic review, using data from 1009 infants, Hutton and Hassan [10] found the risk of developing polycythemia slightly higher in neonates allocated to delayed cord clamping at both 7 hours after birth (RR, 3.44; 95% CI, 1.25–9.52; two trials; *n* = 236) and at 24 to 48 hours after birth (RR, 3.82; 95% CI, 1.11–13.21; 7 trials; *n* = 403) but found no significant difference in mean serum bilirubin levels nor an increased risk of neonatal jaundice within the first 24 hours of life associated with late clamping (RR, 1.35; 95% CI, 1.00–1.81). Cord clamping is performed within very short time after birth, at 10–20 seconds at most in our institution both in vaginal and c/s deliveries. For these reasons, we do not think that cord clamping may lead to any masking of the results and do not accept it as a risk factor in our group. 

Delivery mode and type of anesthesia may influence the jaundice risk. Several previous studies comparing c/s and vaginal delivery did not show a difference in hyperbilirubinemia risk [11–13]. However, in some other studies lower bilirubin levels after c/s are reported and are supposedly explained by placental transfusion or timing of cord clamping [14, 15]. We did not observe a statistically significant difference between cord blood, 24 hours' and 48 hours' bilirubin levels of infants born vaginally or with c/s.

Reports investigating the relation between anesthesia type, anesthetic agent, and hyperbilirubinemia have shown different results [5, 13, 16, 17]. Bupivacaine hydrochloride is a safe and efficient agent for maternal segmental epidural anesthesia. However, it may cause neonatal jaundice by placental passage of anesthetic agent which binds to fetal red blood cell membranes and decreases erythrocyte half-life [18]. Gale et al. [14] claimed that high serum bilirubin levels were related with both c/s and epidural anesthesia. Jouppila et al. [17] found no difference at 12th hour, 2, 3, 4, and 5th day serum bilirubin levels of 37 control infants and 43 infants born after epidural anesthesia with bupivacaine. Similarly, Ozcakir et al. [16] have reported no difference at 1st and 5th day bilirubin levels and the frequency of phototherapy between infants born to mothers who received general anesthesia and epidural anesthesia with bupivacaine. Alkan et al. [13] studied transcutaneous bilirubin levels only at the 24th hour of life and reported higher levels in infants born after general anesthesia (sevoflurane) and epidural (levobupivacaine) anesthesia groups than that of spinal anesthesia (bupivacaine) group.

We found no effects of hyperbaric bupivacaine and levobupivacaine on cord blood, 24 and 48 hours' bilirubin levels. To our knowledge, this is the first study in the literature comparing the effects of these anesthetic agents on consecutive early bilirubin levels in healthy infants. 

Initiation time and type of feeding, maternal anesthesia, gestational age, anatomical problems, and some genetic (cystic fibrosis) or gastrointestinal disorders (meconium plug syndrome, meconium ileus, Hirschsprung's disease or small left colon syndrome), may affect the meconium passage time in infants [19–21]. Normally, infants fed within the first three hours of life have their first meconium passage in the following 4 hours [22]. Since 1 gram wet meconium contains 1 mg bilirubin, delayed passage of meconium and decreased frequency of meconium passage may increase enterohepatic circulation and contribute to the development of jaundice [23]. On the other hand, bilirubin levels are not much affected with the usage of glycerin enema which decreases meconium passage time [23–25]. Evaluating 32 infants born vaginally and 30 infants born after elective c/s, vaginally born infants were more acidotic and passed their first stools earlier. Acidosis is another presumed mechanism, and a role for gastrointestinal hormones is suggested to be the possible mediator of increased gastrointestinal motility in these infants [26]. However, mean meconium passage time was similar in infants born vaginally or with c/s in our study. Meconium passage was delayed in general anesthesia group, but bilirubin levels were not related to the first meconium passage time. 

Early initiation of feeding and feeding intervals less than 3 hours have been shown to be related with lower bilirubin levels [22]. Barrett [27] and Felsher et al. [28] have shown that fasting causes a significant elevation of serum bilirubin levels in both normal adults and patients with hepatic disease. Gartner et al. [29] documented that the enhanced enterohepatic circulation of bilirubin is a major factor in the pathogenesis of fasting-induced hyperbilirubinemia. Early breastfeeding jaundice occurs as a result of insufficient human milk which is mostly due to late initiation and malpractices of breastfeeding [30]. Bertini et al. [31] showed the important role of fasting rather than the type of feeding in the pathogenesis of neonatal hyperbilirubinemia, although breastfeeding per se does not seem to be related to the increased frequency of neonatal jaundice in the first days of life. We have not observed a direct relationship between timing of first feeding and first 48 hours' bilirubin levels. However, delayed initiation of breastfeeding appeared to be a risk factor for being in the high intermediate risk zone of Bhutani's nomogram [9] at 24th hour, and therefore it may be a significant risk for insufficient milk supply and later hyperbilirubinemia. 

Mixed feeding frequency of our total study group was found as high as 41.6%. However, the frequency of formula support was lower (21.5%) in vaginally delivered infants. Our obstetric ward has high rates (72.5%) of cesarean deliveries, and mothers stay at the postoperative intensive care unit for 24 hours. Separation of mother infant dyad probably cause inadequate breastfeeding and early initiation of formula support by medical personnel. These factors are the reasons of the high mixed feeding rate in our study group. 

Bilirubin levels of the first 48 hours were not significantly different in breastfed and mixed fed groups, and the amount of supplemented formula was also not related with the bilirubin levels. Close followup of infants by medical staff and early formula support when needed may be related to reduce fasting hyperbilirubinemia in our group.

Recently iatrogenic late prematurity rate has increased as a result of multiple pregnancies related with assisted reproductive technologies and increased rates of elective c/s. Being mistakenly considered as term infants, this group of late preterm infants have higher risk for hyperbilirubinemia and rehospitalization due to feeding problems, frequent exposure to several drugs, and inadequate followup [22, 32–34]. In addition, their hepatic bilirubin uptake and conjugation are less developed compared to term infants [35, 36]. Insufficient feeding causes dehydration at various degrees and increases enterohepatic circulation and serum bilirubin load as a result [37]. All these factors contribute to more frequent, more severe, and long-lasting neonatal jaundice in late preterm infants.

However, the 24 and the 48 hours' bilirubin levels were found similar in late preterm and term infants in our study. Late preterm infants were more frequently delivered by c/s and had insignificantly delayed meconium passage but received higher amounts of formula supplementation. The similarity of the first 48 hours' bilirubin levels to term infants may be a consequence of close followup and early nutritional support of this special group infants. Serum bilirubin levels beyond 48 hours and rehospitalization rates which may be higher than term infants were not evaluated in our study. 

In the recent study of Ipek et al. [38], cord blood bilirubin levels below 2.6 mg/dL were found to be associated with lower risk of hyperbilirubinemia and further need of phototherapy. Similarly in our study, a 24 hours' bilirubin level over 75th percentile cut off level of 6 mg/dL in Bhutani's nomogram [9] was correlated with cord blood total bilirubin level and also with first feeding time, but not with feeding type and first meconium passage time. Therefore, early initiation of feeding seems as an important strategy for preventing higher bilirubin levels and potential readmissions. 

The limitations of our study are the restricted duration of followup for only first 48 hours and lack of daily weight measurements to show the degree of physiologic weight loss. The strengths of our study on the other hand are the prospective evaluation of several variables that may influence early bilirubin levels and the high number of late preterm infants' data reported from a single center. Moreover, this is the first study that evaluated the effect of spinal anesthetic agent hyperbaric bupivacaine on early neonatal jaundice.

In conclusion, our study demonstrated that mode of delivery, existence and type of anesthesia, feeding modalities, and first meconium passage time had no effect on bilirubin levels during first 48 hours in healthy neonates. Term and late preterm infants had similar early bilirubin levels probably due to our close followup and feeding support policy. First feeding time and high cord bilirubin levels were the most effective factors indicating a high risk for hyperbilirubinemia on Bhutani's nomogram at 24 hours of life. As the secondary acquisitions of our study, we found that vaginally delivered mothers initiated breastfeeding earlier and needed nutritional support for their babies less frequently than mothers delivered with c/s, and meconium passage was delayed after general anesthesia although unrelated to bilirubin levels.

## Figures and Tables

**Table 1:** Demographic characteristics of study infants.

Variable	Number (%)
Gender^‡^	
Female	172 (44.3%)
Male	216 (55.7%)
Fetal growth^‡^	
SGA	10 (2.6%)
AGA	371 (95.6%)
LGA	7 (1.8%)
Delivery mode^‡^	
Vaginal	104 (26.8%)
C/S	284 (73.2%)
Anesthesia type^‡^	
General	39 (13.7%)
Epidural	23 (8.1%)
Spinal	222 (78.2%)
Feeding type^‡^	
Breastfed	227 (58.5%)
Mixed	159 (41%)
Formula	2 (0.5%)
Gestational age^†^ (week)	38.0 (2)
Birth weight^†^ (gr)	3150.0 (695)
Length^†^ (cm)	50.0 (3)
Head circumference^†^ (cm)	35.0 (2)
APGAR score at 1 minute^†^	8.0 (1)
APGAR score at 5 minute^†^	9.0 (1)
Blood group incompatibility^‡∗^	
Mother: Rh (−)/Infant: Rh (+)	25 (6.44%)
Mother: 0/Infant: A or B	55 (14.17%)
Mother: A/Infant: B or AB	25 (6.44%)
Mother: B/Infant: A or AB	13 (3.35%)

^‡^Data presented as number (%).

^†^Data presented as median (interquartile range).

*Hemolytic disease was excluded.

**Table 2:** Relationship of delivery modes and anesthesia types with first feeding, first meconium passage times, feeding types, and bilirubin levels.

				C/S			
	Vaginal (*n*: 104)	C/S (*n*: 284)	*P*	General anesthesia thiopental (*n*: 39)	Epidural levobupivacaine (*n*: 23)	Spinal hyperbaric bupivacaine (*n*: 222)	*P*
First feeding (hour)^†^	0.50 (0.5) [0.1–3]	1.50 (1) [0.2–3.0]	**0.00**	2.00 (1) [0.4–3.0]	2.00 (1) [0.5–3.0]	1.00 (1) [0.2–3.0]	0.20

Breastfeeding^‡^	77.9 (*n*: 81)	50.3 (*n*: 142)		41.0 (*n*: 16)	56.5 (*n*: 13)	52.7 (*n*: 117)	
Mixed feeding^‡^	22.1 (*n*: 23)	49.2 (*n*: 139)	**0.00**	56.4 (*n*: 22)	43.5 (*n*: 10)	46.8 (*n*: 104)	0.29
Formula^‡^	0 (*n*: 0)	0.5 (*n*: 1)		2.6 (*n*: 1)	0 (*n*: 0)	0.5 (*n*: 1)	

Meconium passage (hour)^†^	6.00 (7) [0–26]	6.00 (9) [0–36]	0.50	12.00 (8) [0–36)	5.00 (6) [1–24)	6.00 (7) [0–28]	0.00*

Cord bilirubin^†^ (mg/dL)	1.60 (0.7) [0.3–4.5]	1.60 (0.6) [0.5–4]	0.62	1.70 (0.9) [1–3.5]	1.50 (0.8) [0.9–2.9]	1.60 (0.6) [0.5–4.0]	0.70

24 hours bilirubin^†^ (mg/dL)	4.20 (2.1) [0.9–9]	4.20 (2.1) [0.1–10.6]	0.86	3.70 (2.2) [1.5–8]	4.30 (2.2) [1.8–7.9]	4.20 (2.1) [0.1–10.6]	0.52

48 hours bilirubin^†^ (mg/dL) (median)	6.50 (2.9) [1.8–14.9]	6.60 (2.5) [0.6–13.9]	0.45	6.50 (2.7) [2.6–12]	6.60 (2.7) [3.4–12]	6.65 (2.4) [0.6–13.9]	0.86

^†^Data presented as median (interquartile range: IQR) (min–max).

^‡^Data presented as number (%).

*The statistical difference is due to general anesthesia (thiopental) (Kruskal-Wallis test).

**Table 3:** Relationship of late preterm delivery with delivery modes, first feeding and first meconium passage times, and bilirubin levels.

	Late preterm (*n*: 120)	Term (*n*: 268)	*P*
C/S^‡^	98 (81.7%)	186 (69.4%)	**0.01**
Vaginal^‡^	22 (18.3%)	82 (30.6%)	

First feeding^†^ (hour)	1.00 (1) [0.1–3]	1.00 (1.5) [0.1–3]	0.05

Breastfeeding^‡^	47 (39.2%)	180 (67.2%)	
Mixed feeding^‡^	72 (60.0%)	87 (32.5%)	**0.00**
Formula^‡^	1 (0.8%)	1 (0.4%)	

Anesthesia type			
General^‡^	16 (16.3%)	23 (12.4%)	
Epidural^‡^	9 (9.2%)	14 (7.5%)	0.28
Spinal^‡^	73 (74.5%)	149 (80.1%)	

First meconium passage^†^ (hour)	7.00 (8) [0–28]	6.00 (6) [0–36]	**0.02**

Cord bilirubin^†^ (mg/dL)	1.60 (0.6) [0.8–4]	1.60 (0.6) [0.3–4.5]	0.55

Bilirubin, 24 hours (mg/dL)^†^	4.05 (2.6) (1.0–10.2)	4.25 (2.0) (0.1–10.6)	0.32

Bilirubin, 48 hours (mg/dL)^†^	6.90 (3.1) [2.7–14.9]	6.50 (2.4) [0.6–13.9]	0.07

24 hours bilirubin >6 mg/dL^‡∗^	18 (15%)	39 (14.6%)	0.87

^†^Data presented as median (interquartile range: IQR) (min–max).

^‡^Data presented as number (%).

*The bilirubin 75th percentile cut off level in Bhutani's nomogram [9].
